# A longitudinal analysis of violence and healthcare service utilization in Mexico

**DOI:** 10.1186/s12939-021-01413-6

**Published:** 2021-03-10

**Authors:** Laura X. Vargas, Therese S. Richmond, Heidi L. Allen, Zachary F. Meisel

**Affiliations:** 1 Penn Injury Science Center and School of Nursing, University of Pennsylvania, PA Philadelphia, USA; 2 University of Pennsylvania, School of Nursing, Philadelphia, PA USA; 3 Columbia University, School of Social Work, New York, NY USA; 4 University of Pennsylvania, Perelman School of Medicine, Philadelphia, PA USA

**Keywords:** Violence, Health service utilization, Health services, Longitudinal, Mexico

## Abstract

**Objectives:**

We analyze the degree to which community violence in Mexico, largely due to organized crime violence, affects health care service utilization.

**Methods:**

This study exploits temporal and geographic variation in monthly county-level homicide rates, matching outpatient service utilization from individual longitudinal measures. Sensitivity analyses test for an age specific concentration of violence, respiratory conditions that are likely unrelated to violence, insurance status and health center availability per capita. We test for distributional responses to violence by urban and rural localities.

**Results:**

The likelihood of service utilization increases by 5.2% with each additional homicide per 100,000. When we include self-reported health conditions in the model, our main coefficient remains significant at 4.5%. We find no added effect to our results from interaction terms for age specific concentration of violence, respiratory conditions, insurance status, or health center availability. A substantial increase of 11.7% in the likelihood of service utilization occurs in localities with > = 100,000 inhabitants, suggesting that service utilization is sensitive to the location of violence.

**Conclusions:**

Results highlight the relationship between and increase in violence at the local level and an increase in health care service utilization. This study is among the first to examine this relationship empirically in Mexico. Future research is needed to shed more light on this relationship and its mechanisms.

**Supplementary Information:**

The online version contains supplementary material available at 10.1186/s12939-021-01413-6.

## Introduction

### Background on violence in Mexico

**Fig. 1:**
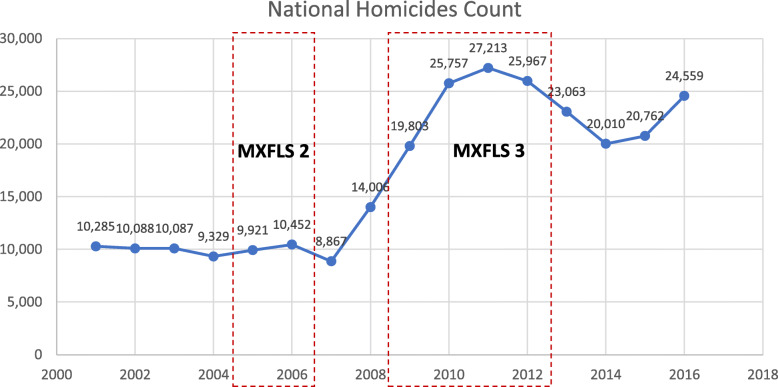
Mexico National Homicide Count 2001–2016

Violence in Mexico has risen to epidemic levels, with more than 200,000 people killed between 2006 to 2018 [1, 2]. During that time, more than 60,000 people disappeared or had gone missing [3]. According to Serrano (2017), the main reasons behind a rapid escalation in violence were threefold. First, during the administration of President Felipe Calderón (2006–2012), Mexico launched an anti-narcotics and drug enforcement effort that confronted criminal groups with public security and military force; this policy in turn contributed to fragmentation and spread of criminal groups into smaller factions as leaders of large criminal groups were targets of the government offensive. Second, while violence accelerated during the period of 2006–2012, Mexico had already undergone a process of militarization of drug enforcement policies that began decades before. Third, drug enforcement policies that began during the Calderon administration not only fragmented large criminal organizations, but also led to heightened competition for territorial and market control among criminal groups. In Fig. 1, we illustrate the rapid rise in homicides in Mexico. The administrations that came after the Calderón administration continued the trend of militarization and drug enforcement policies to confront organized crime, resulting in Mexico recording 2019 as its deadliest year in history (in number of homicides) due to the escalation of violence that has lasted over a decade.

Mexico is a large nation with a total landmass of 1,972,550 km2 and a population of nearly 129 million people. Mexico is the tenth most populated country in the world, with a population density of 66.3 people per square kilometer [4]. Its territory is divided into 32 states, and the 32 states are subdivided into a total of 2456 municipalities (in this manuscript referred to as counties). The number of counties per state varies widely. For example, the state of Oaxaca has 570 counties, while the state of Baja California has five. As a result of Mexico’s geographic and demographic diversity across states, changes in local (county-level) homicide rates will highlight the heterogeneity of violence exposure across counties in our study.

During the period of 2006–2012, crimes such as kidnappings and extortion spread throughout the country, generating fear, stress and anxiety [5–7]. In the period from 2010 to 2015, the number of adults in Mexico who were victims of a crime increased from 18.3 million (roughly 14.2% of the population) to 23.3 million (roughly 18% of the population) respectively [8]. More recent surveys from 2017, report that one in five Mexicans reports being a victim of crime and 93.2% of crimes are not reported or investigated [2, 9]. More than 79% of Mexicans consider their state is unsafe, and half consider their neighborhood is unsafe [9]. More than 64% of Mexicans consider insecurity and violence to be the country’s most pressing social problem, and in 2017 violence accounted for 1.65% of the country’s GDP [9, 10].

Mexico continues to experience civil war-like levels of violence, despite the fact that it is not a country engaged in an official civil conflict or war [11]. Given the epidemic of violence, there is reason to consider that health service utilization might be affected by violence, but it is mostly unknown. This study examines the relationship between changes in individual outpatient service and an increase in community violence.

### Violence and its effects for health and service utilization

Exposure to violence has numerous direct and indirect negative effects for health and health service utilization. There is a negative relationship between violence exposure and health, which may lead to changes in services needed. Further, violence can impact health indirectly through changes in economic activity and local policy. Although the studies presented in this section are evidence of the negative direct and indirect consequences of violence for health, to our knowledge, other studies have not focused on understanding changes in service utilization resulting from violence exposure, which is a plausible pathway impacting short and long-term health.

Direct negative effects of community violence exposure are seen in a variety of health outcomes. Increased cardiovascular health problems [12] and asthma related hospitalizations [13] are related to exposure to community violence. In-utero exposure to terrorist attacks negatively impacted birth weight of newborns in Colombia [14]. One study from Mexico finds that an increase in the county-level homicide rate in Mexico is negatively associated with birth weight of newborns exposed in-utero [15]. In this literature, stress that results from exposure to community violence is identified as a key contributor to worsening health [16]. One study shows that increased community violence leads to worse mental health outcomes and higher levels of risk aversion, the latter of which may prompt changes in health behaviors and daily activities to avoid risk [17]. Changes in health behaviors may influence health outcomes and service utilization.

There are several ways widespread violence can indirectly impact health. Local governments may prioritize public security over public health or other social investments, further affecting population health [10]. While it is a reasonable argument that investing in public security is an upstream strategy to improve health by improving safety, increased violence can generate conditions for increased morbidity and mortality and also weaken the capacity to deal with an increase in adverse health conditions [18]. For example, violence is associated with declines in life expectancy in Mexico for both genders [19]. Promoting security at the expense of investing in the health infrastructure could make individuals more susceptible to the lingering health impacts of exposure to violence.

Evidence from Mexico and the U.S. shows that community violence is related to declines in labor force participation, economic activity and lead to fewer household economic resources available for health and prevention services [20–22]. Violence can hinder preventive health campaigns. For example, in communities in Colombia with high levels of violence, spraying campaigns for mosquito borne diseases were interrupted or not delivered [23]. Community violence can also increase the risk for illness or worsen pre-existing health conditions [13], possibly prompting an increase in demand for services to treat these conditions.

### Possible mechanisms: individual traits and healthcare service utilization

Evidence highlights several demographic contributors to healthcare service utilization. Women are more likely to use primary care services, have lower self-reported health and report fewer years of education and lower income compared to men [24]. Evidence exists of an education-health gradient explaining differences in health outcomes and health behaviors by levels of education [25]. Employment status is both an enabling factor for healthcare service utilization through access to employment-based insurance, but also a limiting factor for outpatient service utilization if employment conditions present a barrier to service utilization [26]. Although employment may be a channel through which individuals and families access healthcare services through employer-based insurance, around 58% of Mexico’s workforce is employed in informal sectors [27]. Most Mexican workers are low-skill and/or low-education [27, 28], and possibly subject to working long hours and/or working in more than one job at a time for subsistence. As a result, it is possible that those who are employed will be less likely to use outpatient services as outpatient services hours may coincide with work schedules.

### Possible mechanisms: Mexico specific contextual contributors to service utilization

Life expectancy in Mexico stagnated between 2000 and 2010 [29–31]. The rise in homicides after 2005 led to a reversal in life expectancy increases among males and a slowdown among females in most states in the period from 2000 to 2010 [29]. The intensity and severity of the increase in homicides during the period of 2005–2010 largely negated any population health gains that resulted from the reductions in other causes of mortality in the decade of 2000–2010 [29]. Life expectancy deteriorated the most among males ages 15–49 after 2005 and has been slow to recover in most Mexican states in the period from 2010 to 2015 [31].

Violence in Mexico is not equally distributed across the country. Much of the organized crime violence that escalated in the period of 2006–2012 was concentrated in urban areas such as Tijuana, Ciudad Juarez, and Matamoros, to name a few [32]. Over time, however, organized crime violence spread to rural areas throughout the country due to their potential for production of primary materials in the drug trade and lower capacity of local law enforcement to combat crime in rural areas [33]. As a result, our empirical strategy considers the difference in distribution of violence in urban and rural localities and its potential to impact healthcare service utilization differently.

In 2003, Mexico implemented a national health insurance program to ensure fundamental access to healthcare for those who were currently uninsured, about 50% of the population. Commonly known as Seguro Popular, the program mostly served those in the informal economy and made publicly funded health protection a universal right as part of a larger reform of the health care system [34, 35]. With an expansion in health insurance in the early 2000s it is possible that healthcare service utilization may have increased.

In 2009, Mexico experienced a rise in the number of influenza cases due to an H1N1 strain [36]. As a result, a population level event such as the influenza outbreak may be directly related to health service utilization. To examine changes in service utilization associated with a large population level shock such as the influenza outbreak, we use self-reported measures of cough and flu available in both waves in our main models and in sensitivity analyses.

We hypothesize that the relationship between violence and health care utilization could go in either direction. On the one hand exposure to community violence influences behaviors to avoid risk (e.g. staying inside, avoiding parts of a community, hypervigilance or limiting youth activity outside) [37–39]. Community violence may have a negative effect on service utilization if adults are deterred by violence from seeking health services or if there are fewer health services available in more violent communities [18]. On the other hand, it is possible that service utilization will increase as a result of violence if increased stress and health behaviors contribute to worsening health [13, 16]. Community violence could also contribute to decreased physical activity, substance use or changes in diet aimed at coping with stress or increasing safety [39–41].

## Methods

### Sample

To assess changes in service utilization related to widespread violence, our study design exploits temporal and geographic variation in individual outpatient service utilization paired with county-level homicide rates from two waves of the Mexican Family Life Survey (MXFLS) longitudinal survey. In our data, all counties have a unique identifier used in the Mexican Census, therefore each county in our data belongs to one state and one state only. The Columbia University Institutional Review Board (IRB) determined this study exempt from review.

The MXFLS is a multi-thematic three wave panel study conducted over a period of ten years. Its baseline survey, MXFLS-1 (2002) collected information on a sample of 35,000 individuals from 8400 households in 150 communities throughout Mexico. The second and third waves, MXFLS-2 and MXFLS-3, relocated and reinterviewed 90% of original households in the baseline sample. The MXFLS survey contains comprehensive sociodemographic data at individual, household and community levels that is representative of the Mexican population [42]. We use individual measures from MXFLS-2 and MXFLS-3, collected during 2005–2006 labeled as a pre-escalation period and during 2009 to 2012 labeled as a during-escalation period, as well as homicide data at the county-level where those individuals reside.

Our sample consists of adults 18 and older: 1) for whom county level homicide rates are available two months before the month and year of interview in MXFLS-2 and MXFLS-3, respectively; 2) who resided in the same municipality during MXFLS-2 and MXFLS-3, and for whom their municipality experienced a non-zero change in the average 2-month homicide rate between MXFLS-2 and MXFLS-3; and 3) for whom the outcome variable of outpatient service utilization is not missing in both waves.

When merging the MXFLS data with the homicide data, we first consider the homicide data for our analysis. Homicide count data are generated through death certificates. Missing values in the homicide count data are likely due to the fact that if no homicides occurred in a given county in a given month and year, a death certificate would not be generated and would be missing. We treat missing values in the homicide count dataset as zero under the assumption that no death certificates with a cause of homicide would be generated if a homicide did not occur. As a result, the homicide data used for this analysis is more heterogeneous because it is not limited to positive non-zero values in any given month and year for the county-level homicide count data.

After merging our datasets, the initial sample consisted of 18,749 adults with individual MXFLS observations for whom 0 and higher homicide rates are available in the two months prior to the month and year of interview in MXFLS-2 and MXFLS-3. We then limit our sample to 8992 individuals who resided in the same municipality during MXFLS-2 and MXFLS-3, and for whom the municipality of residence experienced a non-zero change in the average 2-month homicide rate between MXFLS-2 and MXFLS-3. Our rationale is that by comparing individuals who remained in the same county for both waves we are better able to assign exposure intensity between waves. This choice in our empirical strategy follows Brown (2018), where a similar strategy was used to address endogenous migration associated with violence. Of these, 8559 observations have a non-missing response for our outcome variable (outpatient service utilization) in both MXFLS-2 and MXFLS-3. In our final analytic sample (with non-missing observations for all variables included in our models), we include 8439 individuals from 4547 households located in 117 counties across 16 states.

### Data & Measures

We use individual measures of outpatient service utilization as our main outcome and county-level homicide rates as the main exposure. In the following paragraphs we describe these variables as well as control measures used in our main analyses and covariates used in our sensitivity analyses.

#### Individual outpatient service utilization

The primary outcome drawn from the MXFLS survey is self-reported individual outpatient service utilization. Individual respondents were asked whether they visited a hospital, clinic, or doctor without being hospitalized overnight in the last 4 weeks, where the value of 1 is given if the individual used outpatient services and 0 if not.

#### Homicide rate measure

To match individual health service utilization in our primary data source, we rely on governmental records of homicides as a measure of exposure to violence. Previous empirical studies of the impact of violence on health outcomes in Mexico rely on county level homicides because of its reliability compared to other crime related measures collected at the local level [15, 43]. Homicide data come from the National Health Information System (SINAIS) and the National Institute of Statistics and Geography (INEGI), and we use county-level population data from the National Population Council (CONAPO) to calculate the rate of homicides per 100,000 people. County level homicide data is the most disaggregated level of data that is publicly available. County level homicide rates allow for heterogeneity in levels and timing of violence within all states.

We construct a measure of change in the two-month average homicide as our main independent variable to match responses of service utilization in the past four weeks. To do this, we average the homicide rate of the two months prior to the month of interview as a measure of exposure. This assures, for example, that if an individual was asked about their outpatient service utilization in the past four weeks on the first day of a given month (e.g. December) that there would be a measure of exposure that begins at least one month prior to service utilization (e.g. the average of October and November). We estimate the two-month average homicide rate for MXFLS-2 and MXFLS-3 and then estimate the change in the two-month average homicide rate between both waves as a measure of violence exposure. This measure can be interpreted as capturing the percent change in the probability of outpatient service utilization that results from a one unit increase in the rate of homicide per 100,000 (or for every homicide per 100,000 increase).

To assess the relationship between violence and health service utilization our basic model controls for individual time-invariant and time-varying demographic and health characteristics known to increase outpatient service utilization. Below we describe the main covariates in our models and describe how they were measured:

**Table:** 

Variable	Measurement
Sex in MXFLS-2	We coded female (=1) and male (=0) sex responses
Marital Status in MXFLS-2	We coded whether individual is married (=1) or not (=0).
Education level in MXFLS-2	We group individuals according to their responses to level of education attained: 1 = No schooling, 2 = Elementary School, 3 = Secondary School, 4 = High School, and 5 = High School College/Professional/ Grad.
Age in MXFLS-2	We use a continuous variable for age that ranges from 18 to 97 years old. For sensitivity analyses, we develop a dummy variable where a value of 1 is given to those who are 50 years old and younger, and a value of 0 is given to those older than 50 years old.
Employment in MXFLS-3	We control for individual responses of adults on whether they were employed in the past 12 months (=1) or not (=0)
Insurance status in both waves	We coded having insurance (=1) and not having insurance (=0) responses in both MXFLS-2 and MXFLS-3
Self-reported health conditions in MXFLS-3	We include common chronic health conditions (diabetes, hypertension, heart conditions) and non-chronic health conditions (such as accidents and those associated viral illnesses, such as the flu, cough, body ache, fever and chest pain) as well as stress related conditions (such as depression, anxiety or feeling afraid). We give a value of 1 if the condition is self-reported and a value of 0 if it is not.
Health center availability per 10,000	Geocoded data on the location of health centers in per 10,000 people in each county
Rural and Urban localities	We create a variable for “rural” localities given a value of 1 if the population size is < 2500 inhabitants, and 0 for all other population categories equal or greater than 2500; we do the same for an “urban” variable where 1 is for population size > = 100,000 inhabitants, and 0 for all other categories with less than 100,000.

Following the expansion of health insurance coverage through Seguro Popular described in a previous section, we include insurance status as a control variable to account for its potential to increase service utilization, particularly during the period of rising violence. Proximity to a health center may increase the likelihood of individual outpatient service utilization. The MXFLS asks those adults who have responded “yes” to using an outpatient service whether they know the distance to the service provider and if yes, to provide an estimated distance in kilometers. Unfortunately, only a fraction of individuals who responded positively to using services provided a distance in kilometers to the health center. The self-reported measure is endogenous to individuals who have used services and creates a problem of missing values for all observations in our sample.

Considering the weakness of this measure, we use the earliest available geocoded data on the location of health centers from the year 2013 provided by the Mexican Ministry of Health public data to develop a measure of health center availability per 10,000 people in each county [44]. Although MXFLS-3 data collection takes place between 2009 and 2012, we were not able to find earlier geolocated data of healthcare center locations, thus we assume that the availability of health centers will not have changed much between 2012 and 2013. We map the location of each health center and estimate the number of health centers available per 10,000 individuals in each county. This measure allows us to account for availability of health services at the population level, despite differences in population density between urban and rural localities.

**Fig. 2:**
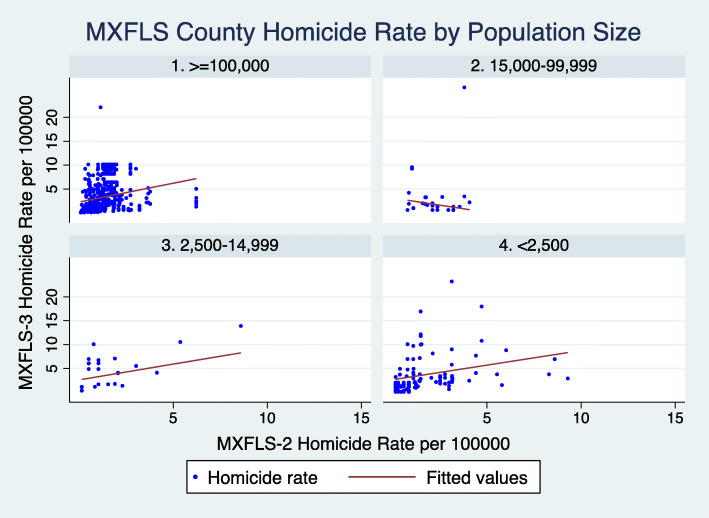
Homicide rate distribution in MXFLS Counties by Locality Population Size

Given existing differences in population density, it is possible that violence may be distributed differently in urban and rural localities, even if they are within the same county. MXFLS adopts classifications developed by the Mexican Census to differentiate between rural localities (those with < 2500 inhabitants) and urban localities (those with 2500-14,999; 15,000-99,999; and those with > = 100,000 inhabitants) within each county. In Fig. 2 of our results we demonstrate a higher concentration of violence in the most urban (> = 100,000) and rural localities (< 2500).

### Analysis

We use mixed-effects logistic regression models to examine the association between violence and outpatient service utilization. We choose this strategy because a fixed-effects logistic regression model is informative of an average association of violence and individual service utilization across counties. But patterns of violence may vary by county or adults within the same household may be more similar to each other in their responses to violence than those across counties. To address this heterogeneity, we use mixed-effects models where county within each state, and household within each county are allowed a separate intercept in the model [45]. Our decision for this modeling structure takes into account: 1) that violence will vary between counties, and thus between states; and 2) that our data consists of individuals who may be in the same household.

The main results of the predictors in the mixed-effects model can be interpreted as individual fixed-effects estimates of the average outpatient service utilization per a one unit increase in the county-level homicide rate change (a one unit increase in the homicide rate change is equal to an increase of 1 per 100,000 average homicide rate). Because fixed-effects are estimated in the model, the random effects for county and household are modeled as deviations from the fixed-effect with a normally distributed variance and a mean of zero. As a result, the primary coefficient reflects an average effect on individual outpatient service utilization over time, for individuals nested within households, within counties.

In the hierarchy of our mixed models, we use counties as the highest level of grouping in our data because county homicides are our measure of exposure and all counties are uniquely associated with a state. We use Stata 16 software for our analysis, which provides a value to assess the improvement of using a mixed effects model over a linear logistic regression model. Stata performs a likelihood ratio (LR) test that compares the fitted mixed model to standard regression with no group-level random effects. If the difference is statistically significant, then the less restrictive model (the one with group-level random effects) is said to fit the data significantly better than the more restrictive model [46].

## Results

Figure 1 shows the total national homicide count over time and as well as the timing of each of the two last two MXFLS waves. Our study calculates the monthly county-level homicide rates by 100,000 people for the years 2004–2013, we show in Fig. 1 how timing of MXFLS-2 and MXFLS-3 waves that are the focus of this analysis overlap with the rising homicide count over time.

Figure 2 shows the distribution of homicides in MXFLS counties by each of the four population size categories used in MXFLS. These categories are also used in the Census to distinguish urban from rural localities. Figure 2 demonstrates a concentration of homicides in urban localities of at least 100,000 people and rural localities of less than 2500 people.

Table 1 shows demographic and health characteristics of adults in MXFLS-2 and MXFLS-3. We test for significant differences in proportions of matched pairs using McNemar’s test to test for changes within individuals over time. The mean age in MXFLS-2 is 42 years old, with 59% of the sample being female, 41% living in rural areas, and education levels increasing slightly from MXFLS-2 to MXFLS-3. Adults are significantly more likely to use outpatient health services, and to have insurance in MXFLS-3. Outpatient service utilization increases by 4.14% from MXFLS-2 to MXFLS-3. The number of those with insurance increased from 50.2% in MXFLS-2 to 64.3% in MXFLS-3, likely due to the rollout of Seguro Popular. As seen in Table 1, significantly more adults were diagnosed with diabetes and hypertension and reported having the flu and a cough in MXFLS-3 compared to MXFLS-2. We observe a decrease in the prevalence of depression from MXFLS-2 to MXFLS-3. We were not able to find an empirical explanation for this change. However, one study conducted in Mexico City observes a similar trend as the one observed in our data and highlights that depression became one of the top ten reasons (eighth) for seeking outpatient services in 2006 [47].

**Table 1:** Individual Demographic and Health Characteristics

	1	2	
	MXFLS-2	MXFLS-3	Prob > chi2
	Sample *N* = 8559	Sample N = 8559	*P*-value
Mean homicide rate (per 100,000)	1.1	2.5	0.000*
Outpatient service use (%)	11.6	15.2	0.000*
Mean Age (years)	42.5	46.7	
Female (%)	59.2		
Married (%)	59.5	60.8	0.000*
Worked in last 12 mos. (%)	57.7	51.5	0.756
Rural (%)	41.0	41.0	
Has Insurance (%)	50.1	64.4	0.000*
Education Level (%)			
No schooling	10.3		
Elementary School	42.6		
Secondary School	24.0		
High School	13.1		
College/Professional/ Grad	9.7		
Ever diagnosed with (%):			
Diabetes	6.8	11.2	0.000*
Hypertension	10.7	15.0	0.000*
Heart Disease	2.2	2.5	0.213
Ever had serious accident	8.2	10.4	0.000*
Last 4 weeks had/felt (%):			
Flu	16.2	24.3	0.000*
Cough	12.2	18.3	0.000*
Stomach Ache	12.2	12.5	0.494
Headache	27.4	29.4	0.001*
Fever	6.4	8.6	0.000*
Body ache	19.2	21.5	0.000*
Chest pain	6.8	8.8	0.000*
Depressed	32.2	29.7	0.000*
Nervous/Anxious	33.4	31.3	0.001*
Felt Fear	22.1	25.0	0.000*

McNemar’s test of marginal proportions in matched pairs; ^*^ *p =* < 0.005

Mixed-effects logistic regression results show a 5.21% increase in the likelihood of service utilization associated with each additional homicide per 100,000 individuals in the county from MXFLS-2 to MXFLS-3 (Table 2, Model 1). The observed increase in service utilization is significant after controlling for age, sex, education in MXFLS-2, as well as employment in the previous 12 months in MXFLS-3, and insurance status in both waves. Our estimates account county and household differences across the sample. Results of the LR test are significant and therefore an improvement on a linear logistic regression model.

**Table 2:** Results of Main Model & Health Conditions Model

	Main Models	
	[1]	[2]
Homicide Rate	Main Model [95% CI]	Main Model + Health Condit. [95% CI]
Outpatient service utilization in MXFLS-3	1.0521^**^	1.0431^*^
	[1.0169–1.0884]	[1.0077–1.0798]
Homicide Rate in MXFLS-2	Yes	Yes
Outpatient service utilization in MXFLS-2	Yes	Yes
Has Insurance in MXFLS-2	Yes	Yes
Has Insurance in MXFLS-3	Yes	Yes
Demographic characteristics	Yes	Yes
Health Conditions MXFLS-3	No	Yes
MXFLS-2 County (_cons)	.1674^***^ [.0956–.2932]	.1676^***^ [.0953–.2948]
MXFLS-2 County >Household (_cons)	.6967^***^ [.4440–1.0932]	.6710^***^ [.4125–1.0916]
Number of Counties	117	117
Number of Households	4547	4546
Observations	8439	8438

Demographic characteristics: age, sex, marital status, education, employment & rural locality

Health conditions: diabetes, hypertension, serious accident, flu, cough, fever, body ache, chest pain, feeling depressed, feeling anxious, feeling afraid

Exponentiated coefficients; Standard errors in parentheses; Integration Points = 10

^*^ *p =* < 0.05, ^**^ *p =* < 0.01, ^***^ *p =* < 0.001

Model 2 in Table 2 adds self-reported individual health conditions, which include ever being diagnosed with diabetes and/or hypertension and ever having had a serious accident. They also include a report in the past four weeks of having: the flu, a cough, a fever, body ache, chest pain, having felt depressed, anxious or nervous and felt more fear in the past four weeks. After including self-reported health conditions as covariates in our model, there is a significant 4.31% increase in outpatient service utilization.

Full models 1–6 available in the Table 1A, include our main models [1, 2] as well as models with interaction terms testing for age specific effects on adults 50 and younger, respiratory conditions, insurance status, and availability of health care centers per county. The coefficients of interest in Models 3–6 in Table 1A are the interaction terms. None of the interaction terms in Table 1A yield significant results. However, the main coefficients remain significant in all of the models. In which case, we observe little heterogeneity in service utilization related to an increase in the homicide rate across our sensitivity analyses.

Table 3 presents the main results of the distributional effects of violence by urban localities of at least 100,000 people and rural localities of less than 2500 people, which concentrate a majority of homicides in MXFLS counties. We find that there are differences in outpatient service utilization associated with violence in urban areas (both coefficients of the interaction terms in Models 7 and 8 of Table 5 are significant). Specifically, for each additional homicide per 100,000 in urban localities with population > =100,000 there is an increase of 11.76% in the probability of using health services (Model 7) and that probability increases to 12.14% after adjusting for self-reported health conditions (Model 8). In rural localities of less than 2500 people, the interaction terms are not significant (Models 9 and 10, Table 2A), though notably the interaction term coefficients change direction, indicating that violence is adversely related to healthcare service utilization in rural areas though not significantly.

**Table 3:** Results of Main Model & Health Conditions Model by Urban Localities (> = 100,000 people)

	Urban Localities (> = 100,000 people) [95% CI]	
Homicide Rate	[7]	[8]
Outpatient service	1.0367	1.0259
	[.9980–1.0768]	[.9864–1.0669]
Urban* HomRate	1.1176^*^ [1.0222–1.2218]	1.1214^*^ [1.0238–1.2251]
Urban	.9922 [.7335–1.3422]	.9356 [.6875–1.2733]
Rural	Yes	Yes
Homicide Rate in MXFLS-2	Yes	Yes
Outpatient service utilization in MXFLS-2	Yes	Yes
Has Insurance in MXFLS-2	Yes	Yes
Has Insurance in MXFLS-3	Yes	Yes
Demographic characteristics	Yes	Yes
Health Conditions MXFLS-3	Yes	Yes
Homicide Rate in MXFLS-2	No	Yes
State (_cons)	.0363*** [.0075–.1756]	.0636^***^ [.0184–.2195]
State>MXFLS-2 County (_cons)	.1104^***^	.0937^***^
	[.0554–.2199]	[.0431–.2035]
Number of States	16	16
Number of counties	117	117
Observations	8439	8438

Demographic characteristics: age, sex, marital status, education, employment & rural locality

Health conditions: diabetes, hypertension, serious accident, flu, cough, fever, body ache, chest pain, feeling depressed, feeling anxious, feeling afraid

Exponentiated coefficients; Standard errors in parentheses; Integration Points = 10

^*^ *p =* < 0.05, ^**^ *p =* < 0.01, ^***^ *p =* < 0.001

## Discussion

This study finds an overall increase in the probability of outpatient health service utilization of 5.21% is associated with a one unit increase in county-level homicide rates in Mexico. This is of concern given the increase in homicide rates between MXFLS-2 and MXFLS-3. We can interpret from our main results that the probability of outpatient service utilization associated with violence more than doubled in our sample. Our results also indicate that the distribution of violence in urban and rural localities is differentially associated with healthcare service utilization. Urban localities account for a concerning increase of 11.76 – 12.14% in the probability of service utilization in response to violence per a unit increase in the homicide rate. This means that individuals in large urban areas will increase their probability of service utilization by roughly one quarter or 25% given the average observed homicide rate in our sample during MXFLS-3.

We explored the possibility that other contextual factors that occurred during the study period might drive healthcare service utilization through several sensitivity tests. Beginning with insurance, it is possible that the expansion of health insurance could drive service utilization. Due to the timing of the *Seguro Popular* universal health insurance program it seems unlikely that rising violence would have affected enrollment. It is also possible that healthcare service utilization increased with *Seguro Popular*. Prior evidence in the U.S. suggests that health care service utilization increases through Medicaid coverage^25^, though one randomized early study finds that Seguro Popular did not drive an increase in service utilization in Mexico^26^. We conclude that it is possible that an increase in health service utilization was impacted by the *Seguro Popular* program and our analysis shows that violence increase variations did not impact this overall trend. Seguro Popular expanded access to insurance for Mexicans who did not have access before. Regardless of expanded access to insurance, Mexico consistently ranks among the lowest among OECD nations in health investments as a share of GDP and crucial infrastructure metrics such as number of doctors and nurses available per 1000 people [48, 49]. Thus, our results underscore the need for making outpatient services more accessible in high violence places.

It is possible that adults experience an increase in stress or other stress related outcomes and seek primary care services because they are more available than specialized mental health services. Our results support evidence from other studies where individuals increased their preventive service use when violence increased, with stress identified as both a mechanism for worse health and protective health behaviors [16, 43]. Prior evidence suggests that exposure to violence is associated with increased hospital visits for asthma, anxiety, substance use, myocardial infarction [16] and with an increase in mental health and substance use problems [50]. While our study did not find added effects of violence for the cold and flu, it does support the overall notion that violence is associated with service utilization among individuals with a variety of health conditions.

To further contextualize stress as a potential driver of the relationship between violence and health service utilization we turn to evidence from the relationship between violence and risk aversion outcomes in Mexico. Brown et al. (2018) find that risk aversion spreads as violent crime increases locally. They point to larger effects among individuals in the middle and upper ranges of the SES gradient, a population that is also more likely to be located in urban areas. It is possible that risk aversion may also influence decisions to seek care in response to violence, a possible area for future research.

We demonstrate that health service utilization related to violence is concentrated in urban areas = > 100,000 inhabitants. The distributional responses to violence in our results are consistent with Torche & Villarreal (2014) who report that low-SES mothers in urban locations exposed to violence in Mexico engage in protective health enhancing behaviors through increased prenatal care visits. The authors suggest increases in stress or anxiety might be behind this behavioral response. Evidence from Guatemala points to a significant increase in the odds of women and urban dwellers experiencing post-violence mental health outcomes in the post-conflict period [50]. Our results suggest that the higher availability of health care services in urban areas may be perceived as a protective resource in response to rising violence.

Given that violence in Mexico acutely affects a specific age group of adults under the age of 50 years old, we test for any such observed effect in our data. Our study does not find evidence of an added effect of violence on health service utilization of adults 50 and younger. One possibility is that younger, working age adults use services less because they are healthier or face work schedule constraints. It is also possible that our data may not reflect more direct non-fatal injury consequences of violence because: 1) violence during our study period was deadlier, which is a possibility based on prior studies [29–31]; or 2) our data on outpatient services may not capture these events with sufficient precision. Future research could explore how violence-related health outcomes (such as non-fatal injury) relate more directly to health service utilization in Mexico.

### Limitations

Outpatient service data relied on individual report of service use. Adults may not recall services used over the four weeks prior to the interview, but it is unlikely that recall errors are systematic enough to introduce bias. Additionally, the specific reason for outpatient service utilization is not reported. Therefore, this analysis is unable to provide a more detailed conclusion on how community violence impacts health or reasons for seeking care.

To study changes in healthcare service utilization over time, we use individual longitudinal outcomes. We match individual level data with county-level homicide rates as a measure of exposure to violence. Homicide data are only available at the county level, therefore a lack of a more granular measure of the homicide rate to match individual level outcomes is a limitation of this study. Our models estimate the intensity of county-level violence experienced by individuals who did not migrate to a different county between waves. As a result, our estimates are internally valid, but cannot be considered externally generalizable to a broader population that may have migrated as a result of local violence in Mexico or to other contexts of violence outside of Mexico.

We have insufficient data to provide an unbiased estimate for distance to healthcare centers based on individual responses of those who did use services. We constructed a measure of healthcare center availability based on earliest available geolocated data of health infrastructure in Mexico in the year 2013. Since data are not available for the period of 2009–2012, which coincides with MXFLS-3 data collection, we assume that the availability of healthcare centers will not have changed sufficiently to alter our results given Mexico’s persistent underinvestment in health infrastructure amongst OECD nations [49].

## Conclusions

This study is one of the first to examine the association between violence exposure and service utilization in Mexico and has important policy implications for the provision and access to health services in contexts of violence. Our findings underscore the importance of making outpatient services more widely available in contexts of violence. While policies such as Seguro Popular have allowed for a larger proportion of Mexicans to be covered for use of primary care services, the availability of crucial healthcare infrastructure in Mexico is lagging compared to other nations. Given the prolonged period of escalating violence in Mexico, policymakers should consider whether broader investments in health prevention, primary care and mental health might be effective in buffering some of the long term (perhaps costlier) impacts of violence on the health of society. One way to do that, for example, is by making trauma informed health services widely available at the primary care level to improve health outcomes in communities exposed to violence.

Widespread violence in Mexico can impact health through various channels. We explore how violence relates to changes in service utilization, as a possible mechanism that may impact health. Violence is a pervasive problem in developing nations such as Mexico and its consequences for service utilization are important to understand. Future studies of health service utilization can focus on the broader repercussions of violence, particularly among economically or otherwise vulnerable populations in urban and rural areas.

## Supplementary Information

**Additional file 1.** Table 1A. Full Models for Main Results & Sensitivity Analyses. Table 2A. A Full Models for Distribution of violence and health service utilization by rural and urban localities.

## Data Availability

The datasets generated and/or analyzed during the current study are available in the MXFLS repository, http://www.ennvih-mxfls.org/english/index.html and in the INEGI repository, https://www.inegi.org.mx/sistemas/olap/proyectos/bd/continuas/mortalidad/defuncioneshom.asp?s=est

## Abbreviations

GDP: Gross Domestic Product
MXFLS: Mexican Family Life Survey
MXFLS-2: Mexican Family Life Survey, Wave Two
MXFLS-3: Mexican Family Life Survey, Wave Three
SINAIS: National Health Information System (Sistema Nacional de Información en Salud)
INEGI: National Institute of Statistics and Geography (Instituto Nacional de Estadística y Geografía)
CONAPO: National Population Council (Consejo Nacional de Población)
IRB: Institutional Review Board
HC: Health Center
