# Supplementary material for: A longitudinal analysis of violence and healthcare service utilization in Mexico
Source: Int J Equity Health. 2021 Mar 10;20:75. doi: 10.1186/s12939-021-01413-6 (PMC7945366; doi:10.1186/s12939-021-01413-6)
Supplement: Supplementary file 1 — Additional file 1. Table 1A. Full Models for Main Results & Sensitivity Analyses. Table 2A. A Full Models for Distribution of violence and health service utilization by rural and urban localities. [file 12939_2021_1413_MOESM1_ESM.docx]

Table 1A. Full Models for Main Results & Sensitivity Analyses

|  | (1) | (2) | (3) | (4) | (5) | (6) |
| --- | --- | --- | --- | --- | --- | --- |
| Homicide Rate | Main Model  [95% CI] | Health Conditions  [95% CI] | =<50 years old  [95% CI] | Respiratory Conditions  [95% CI] | Insurance Status  [95% CI] | # HC per 10k (per county)  [95% CI] |
| Outpatient service utilization | 1.0521^**^ | 1.0450^*^ | 1.0584^*^ | 1.0481^*^ | 1.0621^*^ | 1.0938^**^ |
|  | [1.0169 - 1.0884] | [1.0094 - 1.0818] | [1.0125 - 1.1064] | [1.0095 - 1.0882] | [1.0126 - 1.1140] | [1.0304 - 1.1611] |
| Age | 1.0118^***^  [1.0065 - 1.0171] | 1.0069^*^  [1.0012 - 1.0126] | 1.0159^***^  [1.0079 - 1.0239] | 1.0132^***^  [1.0079 - 1.0186] | 1.0117^***^  [1.0064 - 1.0170] | 1.0118^***^  [1.0065 - 1.0171] |
| Female | 1.5833^***^  [1.3554 - 1.8495] | 1.3673^***^  [1.1604 - 1.6111] | 1.5688^***^  [1.3419 - 1.8341] | 1.5634^***^  [1.337 - 1.8282] | 1.5833^***^  [1.3554 - 1.8495] | 1.5809^***^  [1.3533 - 1.8467] |
| Married | 1.1584^*^  [1.0016 - 1.3398] | 1.1288  [.9725 - 1.3102] | 1.1420  [.9861 - 1.3225] | 1.1626^*^  [1.0043 - 1.3457] | 1.1593^*^  [1.0023 - 1.3408] | 1.1620^*^  [1.0047 - 1.3439] |
| Education:  No schooling | 1.0565  [.8269 - 1.3498] | 1.1186  [.8690 - 1.4400] | 1.0550  [.8257 - 1.3481] | 1.1091  [.8661 - 1.4202] | 1.0541  [.8249 - 1.3469] | 1.0461  [.8188 - 1.3366] |
| Elementary School | 1.0953  [.8193 - 1.4642] | 1.2218  [.9068 - 1.6461] | 1.0860  [.8121 - 1.4522] | 1.1556  [.8622 - 1.5489] | 1.0920  [.8167 - 1.4601] | 1.0774  [.8058 - 1.4406] |
| Secondary School | 1.2710  [.9192 - 1.7575] | 1.4785^*^  [1.0589 - 2.0643] | 1.2666  [.9160 - 1.7513] | 1.3677  [.9861 - 1.8970] | 1.2669  [.9161 - 1.7521] | 1.2468  [.9015 - 1.7245] |
| High School | 1.3466  [.9590 - 1.8906] | 1.7312^**^  [1.2214 - 2.4538] | 1.3372  [.9523 - 1.8778] | 1.4703^*^  [1.0441 - 2.0705] | 1.3417  [.9554 - 1.8842] | 1.3130  [.9346 - 1.8446] |
| Worked in last 12 mos. | .8574^*^  [.7356 - .9994] | .8802  [.7524 - 1.0298] | .8456^*^  [.7244 - .9870] | .8673  [.7435 - 1.0117] | .8571^*^  [.7353 - .9990] | .8576^*^  [.7358 - .9996] |
| Insurance MXFLS-3 | 1.8336^***^  [1.5507 - 2.1680] | 1.7907^***^  [1.5067 - 2.1283] | 1.8399^***^  [1.5559 - 2.1757] | 1.8484^***^  [1.5618 - 2.1877] | 1.8765^***^  [1.5565 - 2.2624] | 1.8247^***^  [1.5431 - 2.1576] |
| Insurance MXFLS-2 | 1.0342  [.8838 - 1.2101] | 1.0548  [.8977 - 1.2394] | 1.0373  [.8864 - 1.2139] | 1.0412  [.8891 - 1.2193] | 1.0322  [.8820 - 1.2079] | 1.0250  [.8760 - 1.1994] |
| Homicide Rate in MXFLS-2 | 1.0028  [.9340 - 1.0767] | 1.0099  [.9400 - 1.0849] | 1.0037  [.9347 - 1.0779] | 1.0012  [.9328 - 1.0746] | 1.0039  [.9347 - 1.0781] | 1.0131  [.9404 - 1.0915] |
| Outpatient Svc. utilization in MXFLS-2 | 1.8457^***^  [1.5267 - 2.2314] | 1.6221^***^  [1.3343 - 1.9719] | 1.8421^***^  [1.5237 - 2.2271] | 1.8259^***^  [1.5087 - 2.2099] | 1.8446^***^  [1.5258 - 2.2301] | 1.8454^***^  [1.5264 - 2.2310] |
| Diabetes |  | 1.5325^***^  [1.2530 - 1.8743] |  |  |  |  |
| Hypertension |  | 1.8049^***^  [1.5082 - 2.1599] |  |  |  |  |
| Heart disease |  | 1.4730^*^  [1.0252 - 2.1163] |  |  |  |  |
| Serious accident |  | 1.5760^***^  [1.2764 - 1.9458] |  |  |  |  |
| Flu |  | 1.1767  [.9685 - 1.4296] |  | 1.4679^***^  [1.1906 - 1.8099] |  |  |
| Cough |  | 1.3047^*^  [1.0616 - 1.6035] |  | 1.4456^***^  [1.1521 - 1.8138] |  |  |
| Fever |  | 1.6285^***^  [1.2953 - 2.0474] |  |  |  |  |
| Headache |  | 1.3616^***^  [1.1570 - 1.6024] |  |  |  |  |
| Body ache |  | 1.2270^*^  [1.0249 - 1.4689] |  |  |  |  |
| Chest pain |  | 1.3178^*^  [1.0527 - 1.6498] |  |  |  |  |
| Felt depressed |  | 1.2098^*^  [1.0197 - 1.4354] |  |  |  |  |
| Felt nervous /  anxious |  | 1.2548^**^  [1.0564 - 1.4903] |  |  |  |  |
| Felt afraid |  | 1.1871^*^  [.9962 - 1.4145] |  |  |  |  |
| Age=<50 |  |  | 1.210 |  |  |  |
|  |  |  | [.9243 - 1.5838] |  |  |  |
| Age=<50* HomRate |  |  | .9908 |  |  |  |
|  |  |  | [.9470 - 1.0366] |  |  |  |
| Cough * HomRate |  |  |  | 1.0611  [.9962- 1.1303] |  |  |
| Flu * HomRate |  |  |  | .9568  [.9027- 1.0140] |  |  |
| Insur MXFLS-3 * HomRate |  |  |  |  | .9864  [.9396 - 1.0354] |  |
| HC per 10k people |  |  |  |  |  | .9636  [.8941 - 1.0384] |
| HC per 10k * HomRate |  |  |  |  |  | .9915  [.9783 - 1.0049] |
| MXFLS-2 County (_cons) | .1674^***^  [.0956 - .2932] | .1688^***^  [.0962 - .2962] | .1677^***^  [.0958 - .2938] | .1655^***^  [.0941 - .2912] | .1678^***^  [.0958 - .2938] | .1619^***^  [.0919 - .2851] |
| MXFLS-2 County >Household  (_cons) | .6967^***^  [.4440 - 1.0932] | .6590^***^  [.4017 - 1.0811] | .6947^***^  [.4424 – 1.0910] | .6906^***^  [.4356 – 1.0948] | .6957^***^  [.4431 – 1.0923] | .6933^***^  [.4411 - 1.0897] |
| Constant | .0288^***^  [.0180 - .0463] | .0167^***^  [.0100 - .02792] | .0217^***^  [.0116 - .0406] | .0213^***^  [.0131 - .0348] | .0285^***^  [.0177 - .0459] | .0317^***^  [.0192 - .0524] |
| # of Counties | 117 | 117 | 117 | 117 | 117 | 117 |
| # of Households | 4,547 | 4,546 | 4,547 | 4,546 | 4,547 | 4,547 |
| Observations | 8,439 | 8,438 | 8,439 | 8,438 | 8,439 | 8,439 |

Exponentiated coefficients; Standard errors in parentheses; Integration Points = 10

^+^ *p=* < 0.1, ^*^ *p=* < 0.05, ^**^ *p=* < 0.01, ^***^ *p=* < 0.001

Table 2A. Full Models for Distribution of violence and health service utilization by rural and urban localities

|  | Urban Localities  (>=100,000 people)  [95% CI] | | Rural Localities  (<=2,500 people)  [95% CI] | |  |
| --- | --- | --- | --- | --- | --- |
| Homicide Rate | (7) | (8) | (9) | (10) | |
| Outpatient service | 1.0367 | 1.0259 | 1.0810^**^ | 1.0707^*^ | |
|  | [.9980 - 1.0768] | [.9864 - 1.0669] | [1.0216 - 1.1438] | [1.0104 - 1.1345] | |
| Age | 1.0118^***^  [1.0065 - 1.0171] | 1.0070^*^  [1.0013 - 1.0128] | 1.0117^***^  [1.0064 - 1.0170] | 1.0070^*^  [1.0012 - 1.0127] | |
| Female | 1.5733^***^  [1.3469 - 1.8379] | 1.3622^***^  [1.1560 - 1.6051] | 1.5778^***^  [1.3505 - 1.8433] | 1.3658^***^  [1.1590 - 1.6096] | |
| Married | 1.1633^*^  [1.0058 - 1.3455] | 1.1316  [.9748 - 1.3136] | 1.1653^*^  [1.0073 - 1.3480] | 1.1332  [.9759 - 1.3157] | |
| Education:  No schooling | 1.0551  [.8259 - 1.3479] | 1.1200  [.8701 - 1.4417] | 1.0511  [.8226 - 1.3431] | 1.1148  [.8658 - 1.4353] | |
| Elementary School | 1.0831  [.8094 - 1.4492] | 1.2207  [.9052 - 1.6463] | 1.0776  [.8052 - 1.4421] | 1.2134  [.8995 - 1.6368] | |
| Secondary School | 1.2382  [.8938 - 1.7154] | 1.4594^*^  [1.0431 - 2.0419] | 1.2408  [.8955 - 1.7193] | 1.4625^*^  [1.0451 - 2.0468] | |
| High School | 1.3008  [.9236 - 1.8321] | 1.7009^**^  [1.1960 - 2.4189] | 1.3030  [.9250 - 1.8355] | 1.7051^**^  [1.1987 - 2.4255] | |
| Worked in last 12 mos. | .85443  [.7330 - .9959] | .8802  [.7523 - 1.0299] | .8547^*^  [.7332 - .9964] | .8791  [.7513 - 1.0287] | |
| Insurance MXFLS-3 | 1.8395^***^  [1.5559 - 2.1749] | 1.7945^***^  [1.5099 - 2.1326] | 1.8330^***^  [1.5503 - 2.1674] | 1.7890^***^  [1.5052 - 2.1264] | |
| Insurance MXFLS-2 | 1.0235  [.8742 - 1.1984] | 1.0500  [.8930 - 1.2347] | 1.0247  [.8750 - 1.1999] | 1.0509  [.8936 - 1.2360] | |
| Homicide Rate in MXFLS-2 | .9908  [.9214 - 1.0654] | .9957  [.9253 - 1.0715] | 1.0060  [.9367 - 1.0804] | 1.0116  [.9412 - 1.0874] | |
| Outpatient Svc. utilization in MXFLS-2 | 1.8504^***^  [1.5306 - 2.2370] | 1.6294^***^  [1.3404 - 1.9807] | 1.8463^***^  [1.5271 - 2.2323] | 1.6247  [1.3362 - 1.9754] | |
| Urban* HomRate | 1.1176^*^  [1.0222 - 1.2218] | 1.1231^*^  [1.0257 - 1.2297] | - | - | |
| Urban | .9922  [.7335 - 1.3422] | .9419  [.6928 - 1.2806] | 1.0625  [.7918 - 1.4258] | 1.0120  [.7498 - 1.3659] | |
| Rural* HomRate | - | - | .9653  [.9023 - 1.0328] | .9664  [.9020 - 1.0355] | |
| Rural | .9586  [.7087 - 1.2968] | .9996  [.7352 - 1.3590] | .9579  [.7055 - 1.3005] | .9957  [.7294 - 1.3593] | |
| Diabetes |  | 1.5291^***^  [1.2505 - 1.8699] |  | 1.5333^***^  [1.2536 - 1.8754] | |
| Hypertension |  | 1.8089^***^  [1.5114 - 2.1648] |  | 1.8031^***^  [1.5065 - 2.1581] | |
| Heart disease |  | 1.4917^*^  [1.0383 - 2.1429] |  | 1.4769^*^  [1.0277 - 2.1224] | |
| Serious accident |  | 1.5782^***^  [1.2782 - 1.9485] |  | 1.5780^***^  [1.2779 - 1.9487] | |
| Flu |  | 1.1718  [.9645 - 1.4235] |  | 1.1758  [.9676 - 1.4288] | |
| Cough |  | 1.3020^*^  [1.0596 - 1.5999] |  | 1.3019^*^  [1.0592 - 1.6003] | |
| Fever |  | 1.6245^***^  [1.2922 - 2.0423] |  | 1.6278^***^  [1.2946 - 2.0467] | |
| Headache |  | 1.3664^***^  [1.1609 - 1.6082] |  | 1.3624^***^  [1.1575 - 1.6036] | |
| Body ache |  | 1.2313^*^  [1.0285 - 1.4741] |  | 1.2273^*^  [1.0251 - 1.4695] | |
| Chest pain |  | 1.3099^*^  [1.0463 - 1.6398] |  | 1.3166^*^  [1.0515 - 1.6484] | |
| Felt depressed |  | 1.2066^*^  [1.0169 - 1.4317] |  | 1.2088^*^  [1.0187 - 1.4343] | |
| Felt nervous /  anxious |  | 1.2516^*^  [1.0537 - 1.4867] |  | 1.2537^*^  [1.0554 - 1.4892] | |
| Felt afraid |  | 1.1882  [.9972 - 1.4160] |  | 1.1884  [.9972 - 1.4162] | |
| MXFLS-2 County  (_cons) | .1682^***^  [.0959 - .2951] | .1740^***^  [.0996 - .30383] | .1643^***^  [.0924 - .2919] | .1717^***^  [.0972 - .3031] | |
| MXFLS-2 County  Household (_cons) | .6880^***^ | .6494^***^ | .6980^***^ | .6606^***^ | |
|  | [.4363 - 1.0849] | [.3932 - 1.0726] | [.4450 - 1.0948] | [.4028 - 1.0833] | |
| Constant | .0298^***^  [.0177 - .0502] | .0171^***^  [.0098 - .0299] | .0292^***^  [.0173 - .0492] | .0167^***^  [.0096 - .02932] | |
| Number of Counties | 117 | 117 | 117 | 117 | |
| Number of Households | 4,547 | 4,546 | 4,547 | 4,546 | |
| Observations | 8,439 | 8,438 | 8,439 | 8,438 | |

Exponentiated coefficients; Standard errors in parentheses; Integration Points = 10

^*^ *p=* < 0.05, ^**^ *p=* < 0.01, ^***^ *p=* < 0.001
